# Patterned Membrane in an Energy-Efficient Tilted Panel Filtration System for Fouling Control in Activated Sludge Filtration

**DOI:** 10.3390/polym12020432

**Published:** 2020-02-12

**Authors:** Aisyah Osman, Normi Izati Mat Nawi, Shafirah Samsuri, Muhammad Roil Bilad, Norazanita Shamsuddin, Asim Laeeq Khan, Juhana Jaafar, Nik Abdul Hadi Nordin

**Affiliations:** 1 Chemical Engineering Department, Universiti Teknologi PETRONAS, Seri Iskandar, Perak 32610, Malaysia; wan_19001650@utp.edu.my (A.O.); normi_16000457@utp.edu.my (N.I.M.N.); shafirah.samsuri@utp.edu.my (S.S.); nahadi.sapiaa@utp.edu.my (N.A.H.N.); 2 Faculty of Integrated Technologies, Universiti Brunei Darussalam, Jalan Tungku Link BE1410, Brunei; norazanita.shamsudin@ubd.edu.bn; 3 Department of Chemical Engineering, COMSATS Institute of Information Technology, Lahore 54000, Pakistan; alaeeqkhan@ciitlahore.edu.pk; 4 Advanced Membrane Technology Research Centre (AMTEC), School of Chemical and Energy Engineering, Faculty of Engineering, Universiti Teknologi Malaysia, UTM Johor Bahru 81310, Malaysia; juhana@petroleum.utm.my

**Keywords:** corrugated membrane, membrane fouling control, membrane bioreactor, tilted panel, activated sludge

## Abstract

A membrane bioreactor enhances the overall biological performance of a conventional activated sludge system for wastewater treatment by producing high-quality effluent suitable for reuse. However, membrane fouling hinders the widespread application of membrane bioreactors by reducing the hydraulic performance, shortening membrane lifespan, and increasing the operational costs for membrane fouling management. This study assesses the combined effect of membrane surface corrugation and a tilted panel in enhancing the impact of air bubbling for membrane fouling control in activated sludge filtration, applicable for membrane bioreactors. The filterability performance of such a system was further tested under variable parameters: Filtration cycle, aeration rate, and intermittent aeration. Results show that a combination of surface corrugation and panel tilting enhances the impact of aeration and leads to 87% permeance increment. The results of the parametric study shows that the highest permeance was achieved under short filtration–relaxation cycle of 5 min, high aeration rate of 1.5 L/min, and short switching period of 2.5 min, to yield the permeances of 465 ± 18, 447 ± 2, and 369 ± 9 L/(m^2^h bar), respectively. The high permeances lead to higher operational flux that helps to lower the membrane area as well as energy consumption. Initial estimation of the fully aerated system yields the energy input of 0.152 kWh/m^3^, much lower than data from the full-scale references of <0.4 kWh/m^3^. Further energy savings and a lower system footprint can still be achieved by applying the two-sided panel with a switching system, which will be addressed in the future.

## 1. Introduction

Membrane bioreactors (MBRs) offer improved effluent quality in order to allow effluent reuse. Such advantages promote worldwide (>200 countries) implementations of MBRs with annual market growth rates of ≈15% [1]. However, membrane fouling remains the major hurdle to boost their competitiveness. It diminishes permeance and system productivity overtime, leading to an increase in operational expenditure mainly for membrane fouling management.

Substantial works have assessed techniques for membrane fouling control [2]. The most common ones through operational-wise are by lowering the operational flux within the sustainable regime, imposing periodical physical and chemical cleanings, or via introducing air [2]. These fouling control means are partly effective, yet their implementation still leads to low fluxes of 18.5 ± 4.8 L/(m^2^h bar), with a peak flux value at 26.6 ± 6.6 L/(m^2^h bar) [1,3]. Improved membrane fouling controls can offer higher membrane productivity and lower operational expenditures associated with it [1].

Some more advanced approaches have been proposed for membrane fouling control. Particle scouring via fluidization has been considered as an energy efficient method, with >90% less than gas sparging [4] thanks to the mechanical scouring of foulant [5], which also imposes damage of membrane integrity [6]. This method is also strongly limited by particle size segregation and uneven fouling control, which eventually propagates the fouling in the long run [7]. Rotating and vibrating membranes have also been seen as fouling control strategies [8,9]. However, these methods are limited by high mechanical energy for moving the module system (membrane panel and water inside it). They also have complex mechanical design and are difficult to scale-up.

Air bubbling is the built-in method provided by most suppliers for fouling control in MBRs [10,11,12]. The air bubbles impose hydrodynamic effects atop the membrane surface to scour-off foulant [13]. The drag force, impact force, and lift force exhibited by the air bubbles create local shear to restrict foulant deposition, and subsequently remove the foulant away from the membrane surface [14].

Air bubbling is largely affected by the bubble size and is highly effective at high velocity under the slug-flow regime [15], and hence a high air pumping energy is required. Despite the high membrane cleaning efficacy, air bubbling has a few inherent limitations [9]. It produces weak shear rates and the impact reaches a “plateau” at a certain air supply. The movement of air bubbles are difficult to control because they tend to reside at the center of the two adjacent vertical membrane panels in the traditional plate-and-frame panels arrangement. Under this situation, only arbitrary impact force from bubbles bouncing between the two panel surfaces helps to scour-off the foulant [15]. Therefore, the conventional system suffers from poor cleaning efficiency and, hence, finding a more energy efficient system is imperative.

Interaction of the air bubbles with the membrane surface can be improved using an ejector-type bubble generator [13]. In this system, the membrane sheet is positioned horizontally with the active side facing downward, and the air bubbles are supplied from underneath leading to full contact of air bubbles with the membrane surface. Another report for flat-sheet ultrafiltration also suggested the advantages of positioning the membrane horizontally to improve system productivity [16]. Recently, we proposed a tilted panel system adopting the same mechanism by maximizing the contact of air bubbles with the membrane surface for membrane fouling control [17]. The system offers 2.7-fold of permeance increment under optimum condition for microalgae filtration and can reach plateau permeance for activated sludge filtration using MBRs [11]. The tilted panel also allows for switching operation in order to enhance the panel productivity [18]. Apart from the plate-and-frame module system, the ceramic tubular module can also be tilted to maximize the contact of air bubbles, and hence improve overall system productivity [19].

Other approaches that have been proposed include exploiting the membrane surface structure (in a form of a patterned membrane surface). The mechanism was based on the similar principle of improving the contact of air bubbles‑to‑membrane [20,21,22,23,24]. The patterned surface enhances the effective filtration area, acts as turbulence promoter, and; therefore, intensifies the contact of air bubble‑to‑membrane [23,25,26,27,28]. The pattern on top of the membrane surface introduces periodic resistance to the axial flow, which promotes mixing in the boundary layer created at the membrane surface due to the formation of fluid eddies, which increase the turbulence, friction, and pressure drop [27,28]. This would be expected to reduce the membrane fouling and improve the hydraulic performance [29,30]. Previous studies focused on exploiting a single approach (i.e., tilting the panel, forming surface corrugation, etc.) to enhance filtration performance and; therefore, obtained a limited degree of improvements. A combined approach, involving more than one approach, can further enhance the throughputs demonstrated in this study.

In this study, we evaluate the tilted panel system coupled with a corrugated membrane to combine the mutual effect of surface patterning and panel tilting in enhancing hydraulic performance. Both a flat membrane (FM) and a corrugated membrane (CM) were first prepared and characterized before assessing, and applied in the vertical and tilted panels. Later, the effects of aeration rate and intermittent aeration for the corrugated membrane (CM) in the tilted panel system were extensively evaluated. Lastly, preliminary assessment of the energy consumption is also provided and compared with the full-scale MBRs data.

## 2. Materials and Methods

### 2.1. Membrane Fabrication, Characterization, and Module Assembly

Both the FM and the CM were fabricated via the phase inversion process by using demineralized water as the non-solvent and the mixture 15 wt.% of polyvinylidene difluoride (PVDF, Arkema, Huston, TX, USA, WM of 300 kDa), 84 wt.% of dimethylacetamide, DMAC (Sigma-Aldrich, St. Louis, MO, USA), 0.5 wt.% of polyethanol glycol (PEG, Sigma-Aldrich, St. Louis, MO, USA), and 0.5 wt.% of lithium chloride (LiCl, Sigma-Aldrich, St. Louis, MO, USA) as the dope solution components. The mixture was stirred at 100 rpm and at 60 °C for 24 h to allow complete dissolution. The solution was then casted atop a non-woven support (Novatexx 2471, Freudenberg-Filter, Weinheim, Germany) using a doctor blade with a wet gap of 0.22 mm. For the FM, the cast film was then immediately immersed into the non-solvent bath. For fabrication of the CM, the steps were similar to that of the FM, except for the introduction of the imprinting step immediately after casting, in order to form corrugation pattern atop the membrane using a net-spacer (47 Mil Parallel: 55%, Sepa^®^ CF Feed Spacer, Sterlitech, Washington, WA, USA) as the master mold according to a method detailed elsewhere [26]. The cast film together with the imprinted spacer were then immersed in the coagulation bath. The prepared membranes were characterized in terms of morphology, contact angle, pore size distribution, porosity, thickness and clean water permeance (CWP) using scanning electron microscopy (SEM, Quanta-250, FEI, Thermo Fisher, Hillsboro, OR, USA), goniometer (OCA 20, Data Physics, Filderstadt, Germany), capillary flow porometer (CFP, Porous Materials, Inc., Ithaca, NY, USA), gravimetrically measured using dry–wet method, digital micrometer (Mitutoyo, Kawasaki, Japan) and cell permeation, respectively. The membrane sheets were stored wet until usage. They were assembled into a filtration panel with an effective area of 120 cm^2^ (one-sided surface of 10 × 12 cm) to allow filtration tests.

### 2.2. Activated Sludge Feed

Activated sludge used as filtration feed in this study was collected from a full-scale aerobic activated sludge process treating domestic wastewater. The system operates at steady-state with effluent chemical oxygen demand, total phosphorus, and total nitrogen of 65 ± 5, 0.9 ± 0, and 19 ± 2 mg/L, respectively. The sludge had mixed liquor suspended solid and mixed liquor volatile suspended solid of 4.1 ± 0.5 and 3.2 ± 0.3 g/L, respectively, slightly lower than the ones in MBRs. The feed was refreshed daily to maintain its condition and to prevent physiological stress of the biomass. This study focused on improving the filterability performance via membrane corrugation and panel tilting and it was not run in the full-MBR mode.

### 2.3. Filtration System Configuration

**Figure 1:**
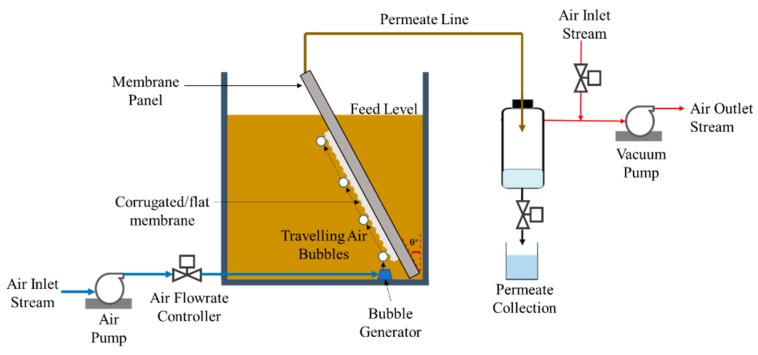
Illustration of tilted panel filtration set-up.

Figure 1 illustrates a custom-built filtration set-up used to assess the filtration performance. The filtration was run under the submerged mode and constant-pressure mode. The filtration panel was submerged in a filtration tank containing activated sludge feed. The panel could be placed in the tank with variable tilting angles. The aeration was provided through a perforated tube underneath the membrane panel. A vacuum pump was used to exert a trans membrane pressure (∆*P*) of −0.2 bar by regulating a control valve. For each filtration, the permeate volume was collected semi-batch-wise under operation cycle of 10 min (9.5 min filtration and 0.5 min relaxation). After recording the permeate volume, it was returned into the tank to maintain its level and other conditions.

### 2.4. Filtration Test

The hydraulic performances were evaluated under different conditions. Firstly, both membranes were assessed under vertical (0°) and tilted (45°) orientation, with aeration at a rate of either 1 L/min or without aeration. It is worth noting that some degree of mixing was performed on the back side of the membrane for test without aeration to maintain the sludge floc under suspension. This preliminary test was conducted to detect the impact of tilting and surface corrugation. After confirming the positive impact of both tilting and corrugation, the hydraulic performance of the CM was later evaluated under variable parameters: Tilting angles (0° and 45°) under constant aeration at a rate of 1 L/min, filtration cycles time (5, 10, 15, and 20 min), intermittent aeration (0, 2.5, 5, and 10 min), and aeration rate (0, 0.25, 0.5, 0.75, 1.0, and 1.5 L/min). Unless otherwise detailed, the filtration cycle time was fixed at 10 min (9.5 min filtration and 0.5 min relaxation); aeration rate was fixed at a constant rate of 1 L/min, and the tests were performed for 2 h at which the queasy steady-state permeance was obtained. After each test, membrane cleaning was performed by soaking the membrane in 1% of sodium hypochlorite (Clorox) solution at 60 °C for 2 h.

Membrane fouling was monitored from the decreasing profile of permeance over time. The flux (*J*, L/m^2^h) and permeance (*L*, L/m^2^hbar) were calculated using Equations (1) and (2).

(1) $$J=\;\frac{V}{A\;\Delta t},$$

(2) $$L=\frac{J}{\Delta P},$$

where *V* is permeate volume (*L*), *A* is effective membrane area (m^2^), and *t* is filtration time (h).

### 2.5. Estimation of Energy Consumption

The energy consumption for the activated sludge filtration was estimated by obtaining relevant data of a full-scale MBR reported elsewhere [31]. The energy consumption of the referenced submerged filtration included influent pumping (*P* *_IN_ of 0.03 kWh/m^3^), permeate pumping (*P* *_P_ of 0.07 kWh/m^3^), coarse bubble aeration (AER * of 0.23 kWh/m^3^), cleaning in place (CIP * of 0.04 kWh/m^3^), and air compression (AIR * of 0.02 kWh/m^3^). The estimated *P*_IN_ for the current system was similar to the *P* *_IN_. However, the *P*_P_ estimation depended on the applied Δ*P* and thus must be predefined. The Δ*P* * and the Δ*P* * were 0.4 bar (as obtained from the reference) and 0.2 bar (as set in the filtration test), respectively. The AER, AIR, and CIP related to the membrane area and thus must be factored in the equation as function of membrane area [32]. The required membrane area in a full-scale system is inversely proportional to the applied fluxes. By considering those factors, the energy consumption of the filtration (*E*, kWh/m^3^) was estimated using Equations (3) and (4).

(3) $$E=P{\;}_{IN}^{*}+\frac{\Delta P}{\Delta P{\;}^{*}}P{\;}_{P}^{*}+\frac{J^{\;*}}{J}\left(AER{\;}^{*}+CIP^{\;*}+AIR{\;}^{*}\right).$$

By inserting the values of Δ*P* *, Δ*P*, and *J* * of 0.4 bar, 0.2 bar (as applied in the experiment), and 28 L/m^2^h, respectively, Equation (3) can be simplified into Equation (4) that correlates the *E* with the applied flux.

(4) $$E=0.065+\frac{8.12}{J}.$$

## 3. Results and Discussion

### 3.1. Membrane Fabrication and Characterization

**Figure 2:**
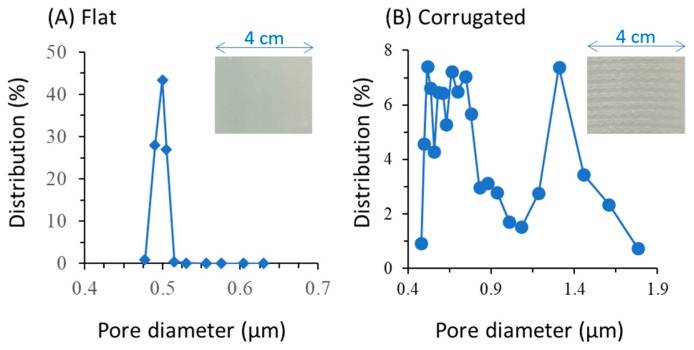
Pore size distribution of (**A**) the flat and (**B**) the corrugated membrane samples. The insets show the picture of the membrane surface.

The introduction of imprinting step was found to be effective in forming surface corrugation on the CM (see inset of Figure 2). The FM has a smooth surface, meanwhile the CM has a rough surface with obvious linear patterns atop it, as further illustrated in SEM images in Table 1. The changes on surface morphology could not be provided because of the challenges in obtaining the SEM image. However, it can be found in an earlier report employing almost a similar fabrication technique [26]. The formation of the corrugation pattern is then expected to increase local mixing and turbulence to scour-off foulant, as later discussed. The results confirm the efficacy of the imprinting technique in forming a surface pattern as reported earlier [23]. It is widely reported that surface corrugation offers improvement for mass transport, reduction in membrane fouling formation, and improved energy consumption [29].

Imposing surface corrugation largely affects the membrane properties as summarized in Table 1. It shows that introducing an imprinting step to form surface corrugation leads to an increase in contact angle (99° ± 2.39° to 106° ± 3.70°), pore size (0.5 ± 0.02 to 0.78 ± 0.10 µm; as shown in Figure 2), porosity (41% ± 6.0% to 65% ± 8.1%), thickness (228 ± 3.21 to 269 ± 5.32 µm), and CWP (550 ± 3.02 to 642 ± 0.32 L/m^2^ h bar). The underlying mechanisms for formation of such structure have been detailed elsewhere [30]. This study focuses only on the mutual effect of surface corrugation and a tilted panel in enhancing hydraulic performance [29]. Nonetheless, it is worth noting that the changes in structural properties due to the imprinting step make it difficult to decouple the sole effect of surface corrugation compounded with other properties on the hydraulic performance [33]. Table 1 shows that the CM is thicker than the FM, due to extra space occupied by the patterned surface, and it is more hydrophobic because of the different step in the fabrication method. Among the properties listed in Table 1, pore size and CWP are the ones with the most prominent impact on hydraulic performance, and they favor the CM. Therefore, when analyzing the filterability results, the focus of the discussion is on the impact of the corrugation, pore size, and CWP.

### 3.2. Effect of Aeration, Corrugation, and Tilting Angle

**Figure 3:**
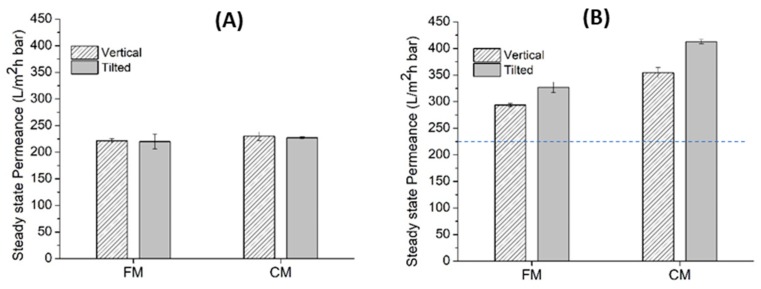
Comparison of the flat membrane (FM) and the corrugated membrane (CM) in the tilted and vertical panels (**A**) without and (**B**) with aeration of 1 L/min. The dashed line indicates the filterability performance of FM without aeration positioned in vertical alignment, used as reference.

The advantage of higher CWP and pore size posed by the CM panel leads to slightly higher permeance, either under the vertical or under the tilted alignments. Figure 3A compares the steady-state permeance of the FM and CM under vertical and horizontal modes without the presence of aeration. Under this configuration, no membrane fouling control method was applied. As a result, no effect of panel tilting is observed. The finding suggests that without appropriate membrane fouling control, advantages of superior intrinsic membrane properties, as shown by the CM in this context, can be overshadowed by the membrane fouling. In other words, the extent of membrane fouling is prominent, as such the fouling resistant impact by the membrane material becomes insignificant.

The superiority of a membrane (in the form of CM) or a system (in the form of tilted panel) can obviously be seen under correct circumstances, as shown by comparing Figure 3A,B, whereby, in the latter, aeration as a mean for fouling control was applied. Figure 3B compares the permeance of the FM and the CM in the tilted and the vertical panels with aeration supply. Two main phenomena are demonstrated; tilting offers higher productivity than the vertical; and the CM has higher permeance than the FM. The impact of aeration is more prominent for the CM than the FM. The permeances of the FM and the CM are 33% and 54%, respectively, higher than the reference system with no aeration under vertical panel (indicated by the dashed-line in Figure 3B). This result suggests that the corrugated surface of the membrane contributes in disturbing the trajectory of the air bubbles and increases flow hydrodynamics near the membrane surface, as suggested by others [26]. It also promotes turbulence, which limits the foulants deposition.

The impact of surface corrugation and aeration is even more pronounced on the tilted system, which are 49% and 87% higher than the reference for the FM and the CM, respectively. This finding demonstrates the efficacy of the combined effect of tilting, aeration, and surface corrugation in enhancing filterability. Tilting the membrane panel improves permeance by maximizing the contact of air bubbles onto the membrane surface and thus improving the scouring impact [17]. In this work, the permeance of the CM under the tilted panel (413 ± 4.04 L/m^2^hbar) is 17% higher than the vertical (354 ± 10.10 L/m^2^hbar), while the permeance of the FM under the tilted panel (327 ± 10.10 L/m^2^hbar) is 11% higher than the vertical (293 ± 3.93 L/m^2^hbar). This finding shows that the impact of panel tilting on the hydraulic performance is more prominent on the CM. The outcomes of these tests lead to a conclusion that the combination of the three parameters (surface corrugation, aeration, and penal tilting) leads to the highest system productivity. The study then focuses on exploring the operational condition for running the CM under either vertical or tilted configurations.

### 3.3. Effect of Filtration Cycle Involving Relaxation

**Figure 4:**
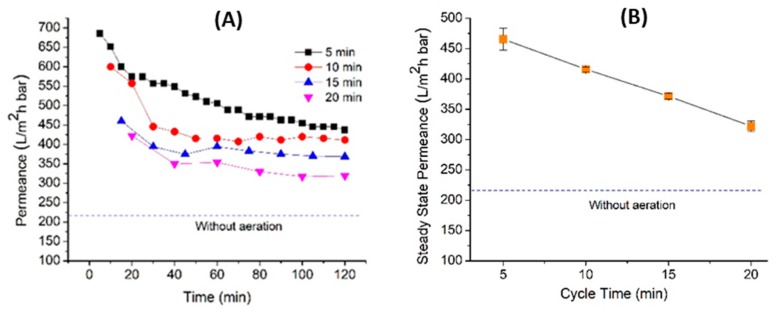
Performance of the corrugated membrane with tilted panel with different cycle times showing (**A**) the permeance as function of time and (**B**) summary of the steady-state permeance.

Figure 4 shows a performance of the CM at different filtration cycles of 5, 10, 15, and 20 min. The relaxation periods correspond to 10% of the cycle period. This test was performed to scrutinize the effect of relaxation period on fouling control. Relaxation is one of the physical cleaning methods performed by temporarily stopping the filtration. Figure 4A summarizes the performance of the membranes as a function of filtration time. Basically, all filtration tests conducted in this study show almost a similar trend, which is high permeance at the beginning of filtration but then drastically dropping due to fouling effect, before becoming constant at the end of filtration.

As shown in Figure 4B, the permeance gradually declines with the prolonged cycle periods, from 465 ± 18.03 L/m^2^ h bar for the 5 min cycle time to 322 ± 8.38 L/m^2^ h bar for the 20 min cycle time. This situation suggests that the membranes suffer from fouling and it is more significant at longer filtration cycles. The periodic relaxation stops the drag force of the permeating fluid and thus allows the release of the accumulated foulants via back-transport. Since a longer cycle requires a longer continuous filtration period, it gives more time for foulant accumulation including irreversible foulant that is poorly removed by the aeration. This causes relaxation to become less effective in releasing the layer of accumulated foulants. At shorter cycle (i.e., 5 min), the relaxation period is shorter but more frequent (i.e., four times more frequent than the 20 min cycle time). Since the filtration period is shorter, less foulants are deposited on the membrane surface. Thanks to a more frequent relaxation mode, the foulants are easily released and scoured off by the air bubbles.

### 3.4. Effect of Aeration Rate

**Figure 5:**
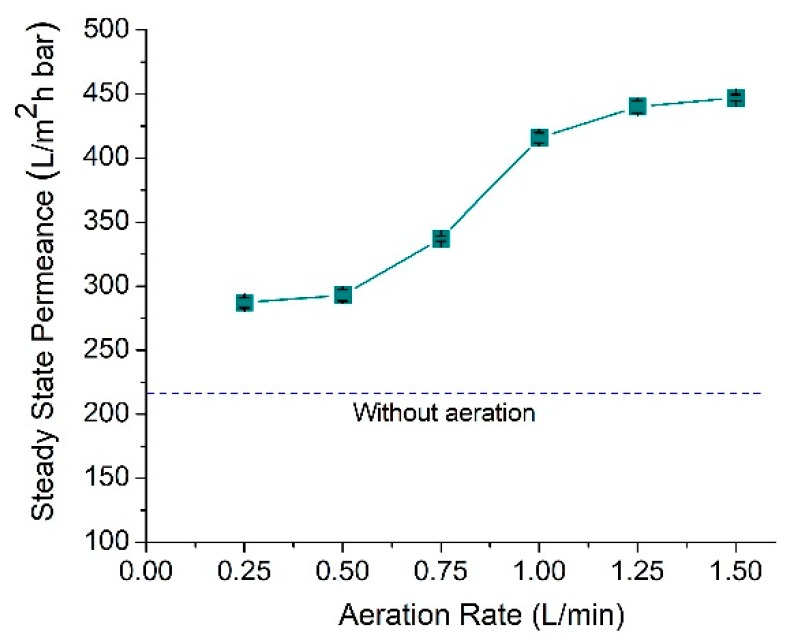
Steady-state permeance of CM as a function of aeration rate.

Figure 5 illustrates the steady-state permeance for the system run at different aeration rates. It shows that the filterability increases with the increment of aeration rate. The results obtained are as expected, since higher aeration rates tend to release more air bubbles with larger sizes and provide more intense contact with the membrane surface to scour off the foulant [34]. A higher aeration rate enhances the scouring impact to scour off the foulant and to prevent its accumulation. Besides, introducing a higher intensity of air bubbles are also expected to induce turbulent flow near the membrane surface, hence more collisions occur between air bubbles and foulants which is beneficial for the foulant removal. The panel tilting and surface corrugation further intensify forces acting on the membrane surface (in the form of impact force and drag force exerted by the bouncing air bubbles on the corrugated surface) [17]. These forces promote efficient foulant removal and inhibit foulant build up, thus enhance the steady-state permeance.

The impact of panel tilting is more prominent at a range of aeration rates of 0.5 to 1 L/min. At a lower aeration rate (i.e., 0.25 L/min), the number of bubbles is too low to impose remarkable impacts on foulant removal, and as the rate increases to a range of 0.5 to 1.0 L/min, an ample number of bubbles are present to impose membrane cleaning. Beyond 1.0 L/min, the impact of aeration rate becomes constant, since the permeance has reached the plateau value of about 440 ± 3.82 L/m^2^ h bar. A plateau permeance is a condition where the reversible fouling rate is very low due to the effective fouling control. Plateau permeance is referred to as the condition where the air bubbles scouring can maintain the membrane free from the accumulation of foulant. Under the plateau permeance condition, improvement of physical cleaning by introducing a higher aeration rate does not significantly affect the permeance. In other words, the number of air bubbles has reached a threshold value where most physical cleaning has been achieved. It means that employing an aeration rate beyond 1.5 L/min might not improve the permeance [35].

### 3.5. Effect of Intermittence Aeration

Intermittent aeration is a process to periodically stop the aeration in order to save energy consumption. It can also be seen as the performance of the tilted panel system operated under switching panel mode to improve panel productivity with alternating aeration [18]. The switching panel is considered beyond the scope of this study. In this study, aeration cycle periods were set at 2.5, 5, and 10 min. An aeration cycle of 2.5 min consists of aeration for 2.5 min followed by no aeration for 2.5 min, and so on. It means that, implementing intermittent aeration offers 50% energy saving for aeration. During the aeration off period, the aeration was introduced on the back side of membrane panel to maintain good mixing of the activated sludge. The 0 min point of the aeration period represents the constant aeration.

**Figure 6:**
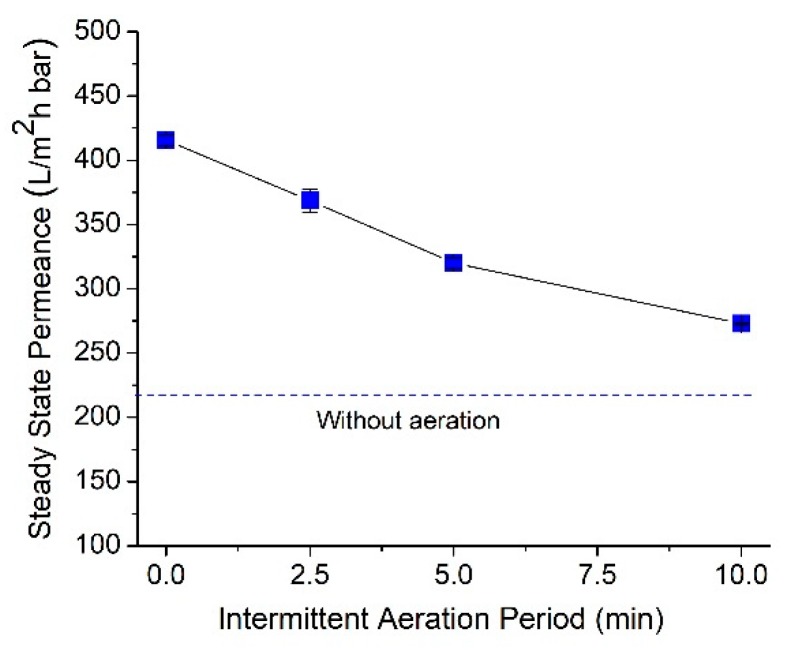
Filterability performance of the corrugated membrane as a function of intermittence aeration period.

Figure 6 shows the negative impact of intermittent aeration on permeance but might lead to substantial energy saving for aeration energy. Figure 6 shows that a longer intermittent aeration cycle does not favor the membrane permeance. When intermittent aeration was increased from 2.5 to 10 min, the steady-state permeances of the membranes drop about 25%. Longer intermittent aeration periods provide more time for the foulant deposition, accumulation, and, most likely, cake layer compaction on the membrane surface. When the aeration is active, the cleaning impact of the air bubbles cannot fully restore the permeance, leading to lower hydraulic performance. On the other hand, in a short intermittent aeration period, the time span of the system with no aeration is rather short for foulant accumulation. Hence, when the aeration is active, the forces exerted by the air bubbles are enough to scour off the deposited foulants. Overall, the results in Figure 6 suggest the application of a short intermittent period to maintain high steady-state permeance. In the short intermittent cycle period, the energy consumption is similar to the longer period, but the frequency to switch on and off the aeration is higher.

The energy consumption when applying the CM can be estimated using Equation (4). The highest permeance of 465.6 L/m^2^ h bar, obtained for the condition of full aeration, corresponds to a flux of 93.1 L/m^2^ h when operated at ΔP of 0.2 bar. Based on Equation (4), the estimated energy consumption is 0.152 kWh/m^3^. This value is 2.6 times lower than the one reported by the reference full-scale MBR of 0.40 kWh/m^3^ [32], and much below the upper threshold of the multiple optimized MBR demonstration plants surveyed by Japan Sewage Works Agency, with specific energy consumptions of <0.4 kWh/m^3^ [32]. The obtained energy estimation is also lower than the one obtained using the optimized tilted panel system involving panel switching of 0.276 kWh/m^3^ [11]. The high energy saving originates from the high flux offered by the CM in the tilted panel system, which is 4.9 times higher than the average flux of the full-scale MBRs [1]. Nonetheless, implementation of the tilted panel system may lead to a larger footprint because of the space required for panel placements. It is also worth noting that there are many factors affecting the accuracy of the estimations. Module train arrangement, feed fouling propensity, and the nature of fouling that leads to variable chemical cleanings are among the factors that can affect the estimation value. The full-scale MBRs’ operational parameters also vary largely [1], which make an accurate estimation difficult. The energy saving can potentially be enhanced under a two-sided panel system involving panel switching, as reported elsewhere [1]. Panel productivity, operational method, and analysis of energy consumption for a switching panel system will be addressed in a future study.

## 4. Conclusions

The patterned surface in the corrugated membrane (CM) was proven to increase local mixing and introduce turbulence near to the membrane surface, which leads to better fouling control. The impact is more profound in a tilted panel system that maximizes the contact of air bubbles and the membrane surface. The combination of surface corrugation, higher air bubbling rate, and panel tilting achieved up to 47% permeance improvement. The CM in the tilted panel system works better when operated under the short filtration–relaxation cycle (i.e., 35% improvement from cycle time of 10 to 2.5 min). Similarly, operation at a longer intermittent aeration cycle leads to lower steady-state permeance. Higher aeration rate improves the steady-state permeance of the membrane significantly most prominently at a range of 0.5 to 1.0 L/min. High permeability associates closely to energy consumption, which was estimated to be 0.152 kWh/m^3^, much lower than data from the full-scale references of <0.4 kWh/m^3^. Overall findings suggest the mutual advantage of surface corrugation and panel tilting in improving overall system productivity. Both surface corrugation and panel tilting intensify the contact of air bubbles to the membrane surface and, hence, offer great potential to be applied in MBRs, or in other liquid filtrations.

## Figures and Tables

**Table 1:** Characteristics of polyvinylidene fluoride membranes.

Properties (Unit)	Flat Membrane	Corrugated Membrane
Pore morphology SEM images	Asymmetric 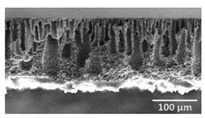	Asymmetric 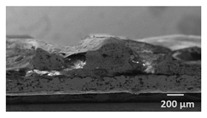
Contact angle (°)	99 ± 2.39	106 ± 3.70
Average pore size (µm)	0.5 ± 0.02	0.78 ± 0.10
Porosity (%)	41 ± 6.0	65 ± 8.1
Thickness (µm)	228 ± 3.21	269 ± 5.32
Clean water permeance (L/m^2^ h bar)	550 ± 3.02	642 ± 0.32
